# A Sensor Fusion Method Based on an Integrated Neural Network and Kalman Filter for Vehicle Roll Angle Estimation

**DOI:** 10.3390/s16091400

**Published:** 2016-08-31

**Authors:** Leandro Vargas-Meléndez, Beatriz L. Boada, María Jesús L. Boada, Antonio Gauchía, Vicente Díaz

**Affiliations:** 1 Mechanical Engineering Department, Universidad Carlos III de Madrid, Avda. de la Universidad 30, Madrid 28911, Spain; lvargas@ing.uc3m.es (L.V.-M.); mjboada@ing.uc3m.es (M.J.L.); vdiaz@ing.uc3m.es (V.D.); 2 Mechanical Engineering-Engineering Mechanics Department, Michigan Tech University, 1400 Townsend Drive, Houghton 49931, Michigan, MI, USA; antonio@mtu.edu

**Keywords:** sensor fusion, roll angle estimation, neural network, linear Kalman filter

## Abstract

This article presents a novel estimator based on sensor fusion, which combines the Neural Network (NN) with a Kalman filter in order to estimate the vehicle roll angle. The NN estimates a “pseudo-roll angle” through variables that are easily measured from Inertial Measurement Unit (IMU) sensors. An IMU is a device that is commonly used for vehicle motion detection, and its cost has decreased during recent years. The pseudo-roll angle is introduced in the Kalman filter in order to filter noise and minimize the variance of the norm and maximum errors’ estimation. The NN has been trained for J-turn maneuvers, double lane change maneuvers and lane change maneuvers at different speeds and road friction coefficients. The proposed method takes into account the vehicle non-linearities, thus yielding good roll angle estimation. Finally, the proposed estimator has been compared with one that uses the suspension deflections to obtain the pseudo-roll angle. Experimental results show the effectiveness of the proposed NN and Kalman filter-based estimator.

## 1. Introduction

Recent developments in vehicle technology have steered the industry towards an increase in vehicle safety, and it is now considered to be one of the key features of a vehicle, even at the initial design stages. One of the main causes of traffic accidents in which heavy vehicles are involved is the loss of lateral stability. Heavy vehicles are prone to roll over since the ratio of the height of the center of gravity to the wheel track is high. The loss of lateral stability is caused when the tire-road contact force on one of the wheels becomes zero under lateral acceleration.

Nowadays, vehicles are equipped with control systems in order to improve their handling and stability. Systems, such as Roll Stability Control (RSC) and Electronic Stability Control (ESC), need to know in advance the expected vehicle behavior during different maneuvers in order to adequately actuate on the vehicle systems [1,2].

RSC systems are based on lateral load transfer, which is directly related to the vehicle roll angle [3]. Therefore, to improve the performance of these systems, an accurate measurement of the vehicle roll angle is needed [4]. The roll angle can be obtained from a dual-antenna GPS, but this is a very expensive technique. For this reason, roll angle has to be estimated [5,6,7]. There are mainly two techniques used for its estimation: integration of information from sensor measurements (sensor fusion) and the usage of a physical vehicle model [8]. Sensor fusion technologies are widely used; they operate by integrating low-cost sensors in many vehicle applications. In principle, the fusion of multisensor data provides significant advantages over single source data. The use of multiple types of sensors may increase the accuracy with which a quantity can be observed and characterized [9]. Doumiati et al. [10] proposed a method to estimate the roll angle using measurements from potentially integrable sensors, such as accelerometers and suspension deflection sensors. If the pitch dynamic effects on roll motion are neglected, the roll angle can be calculated as:

(1) $$\theta =\frac{\left(\Delta_{11}-\Delta_{12}+\Delta_{21}-\Delta_{22}\right)}{2e_{f}}-\frac{m_{v}a_{ym}h}{k_{t}}$$

where $\Delta_{ij}$ is the suspension deflection, $a_{ym}$ is the lateral acceleration, $k_{t}$ is the roll stiffness resulting from tire stiffness and $m_{v}$ is the vehicle weight. Nevertheless, suspension deflection sensors are expensive, so real-time measurement of the roll angle is typically not available for vehicles [11].

For this reason, different algorithms based on the fusion of other types of sensors are proposed. Low-cost GPS and onboard vehicle sensors are also employed [12,13]; however, satellite visibility might be poor in urban and forested driving environments, yielding inaccurate estimations [14].

Rajamani et al. [11] propose a dynamic observer that utilizes the information provided by only a lateral accelerometer and a gyroscope. Nevertheless, the estimation of roll angle has a significant error in transient response. In this algorithm, neither measurements noise nor model noise are considered. Other authors propose several sensor fusion techniques in combination with Kalman filters. A Kalman filter is frequently used for sensor fusion applications because it is an optimal estimator, is straightforward to implement and can be adapted to multiple sensor scenarios [15] and take into account the measurements and model noises. Common applications include vehicle localization [16], estimation of sideslip [17] and roll vehicle angle estimation [14,18].

**Figure 1:**
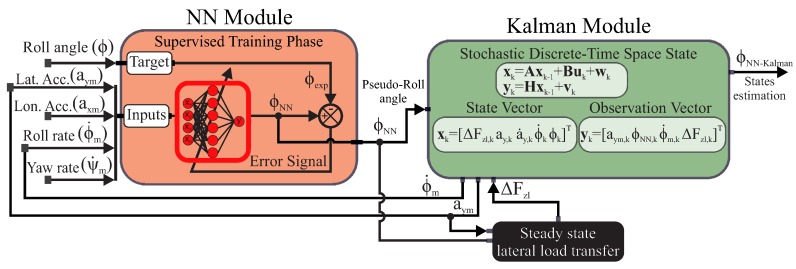
Estimator architecture.

In this paper, we propose a novel estimator based on a Neural Network (NN) combined with a Kalman filter in order to estimate the vehicle roll angle (see Figure 1). Previous work has combined AI-based techniques with a Kalman filter for estimation; however, in our case the IA-based algorithm is based on the improvement of filter performance through the adaptive estimation of the filter statistical information (covariance matrices) [19,20,21]. A difficulty is that uncertainty learning is a difficult and complex process. In this research, we not only estimate the filter statistical information, but also a “pseudo-parameter”, a pseudo-roll angle, which is introduced in the Kalman filter. The NN system estimates a pseudo-roll angle through variables that are easily measured by an IMU, a device that has recently become less expensive. The pseudo-roll angle is introduced in the Kalman filter in order to filter out noise and minimize the variance of the estimated norm and maximum errors.

The rest of the paper is organized as follows. In Section 2, a description of the estimator architecture is given. The estimator architecture is formed by two modules: the NN module and the Kalman module. The former estimates a pseudo-roll angle, and the latter filters the noise and minimizes the norm and maximum errors. Section 3 presents the estimator results, both with simulated and experimental scenarios. A discussion of these results is presented. Finally, Section 4 concludes this paper.

## 2. Vehicle Roll Angle Estimator Architecture

The architecture of the proposed estimator is given in Figure 1. The architecture is formed by two modules: the NN module and the Kalman module. The NN module estimates a pseudo-roll angle through data, such as the lateral accelerometer signal, $a_{ym}$, the longitudinal accelerometer signal, $a_{xm}$, the yaw rate sensor signal, ${\dot{\psi }}_{m}$, and the roll rate sensor signal, ${\dot{\phi }}_{m}$. These signals are easily measured by an IMU, the cost of which has decreased in recent years. The pseudo-roll angle is fed into the Kalman module in order to filter noise and minimize the maximum and norm errors.

The proposed method has the advantage of taking into account the vehicle non-linearities, thus yielding a good roll angle estimation.

### 2.1. NN Module

**Figure 2:**
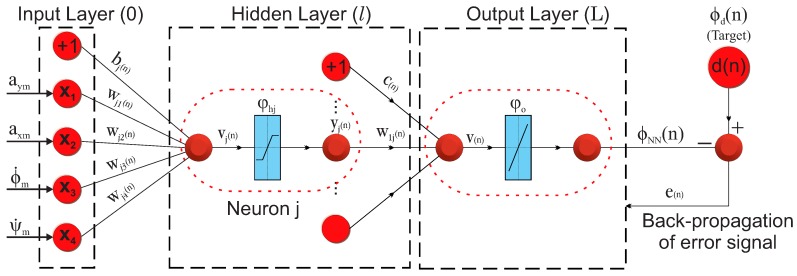
Neural network architecture.

The NN module uses artificial neural networks to estimate roll angle. The preliminary reconstruction of the roll angle from an NN-observer is used as a “pseudo-measurement” in the Kalman filter. The proposed model employs a Back-Propagation (BP) algorithm, which is one of the most widely-used methods for training a neural network. The architecture of the BP neural network is shown in Figure 2. The NN has a single hidden layer with 15 neurons, four inputs (the lateral accelerometer signal, $a_{ym}$, the longitudinal accelerometer signal, $a_{xm}$, the yaw rate sensor signal, ${\dot{\psi }}_{m}$, and the roll rate sensor signal, ${\dot{\phi }}_{m}$) and one output (the vehicle roll angle, ϕ).

The network parameters, synaptic weights and bias, are adjusted with the error signal. The error signal, *e*, is defined as the difference between the desired response, $\phi_{d}$, and the estimated response of the network, $\phi_{NN}$. The learning process is maintained on an epoch-by-epoch (an epoch is one complete presentation of the entire training set during the training process) basis, until the synaptic weights and bias levels of the network stabilize and the average squared error over the entire training set converges to some minimum value [22].

The sequential mode of training is divided in into five stages. The first stage, called initialization, employs random and small values close to zero for the weights and biases in both the hidden and the output layer. In the second stage, the training patterns ((x(1),d(1)),…,(x(n),d(n)) are presented to the NN. The vector $x\left(n\right)=\left\{x_{1}\left(n\right),x_{2}\left(n\right),x_{3}\left(n\right),x_{4}\left(n\right)\right\}$ represents the input vector, and d(n) represents the desired response, respectively, at iteration *n*. In the third stage, each hidden layer neuron calculates the sum of the weight inputs; it applies an activation function $\varphi_{hj}$ and sends their results to the output layer. The output of the *j*-th hidden layer neuron at iteration *n* is calculated as follows:

(2) $$y_{j}\left(n\right)=\varphi_{hj}\left(v_{j}\left(n\right)\right)$$

where $v_{j}\left(n\right)$ is the weighted sum of the inputs of the network in the *j*-th hidden layer neuron:

(3) $$v_{j}\left(n\right)=\sum_{i=1}^{4}w_{ji}\left(n\right)x_{i}\left(n\right)+b_{j}\left(n\right)$$

where $b_{j}$ is the bias.

These signals are transmitted to the output layer. The output is calculated as follows:

(4) $$\phi_{NN}\left(n\right)=\varphi_{o}\left(v\left(n\right)\right)$$

where v(n) is the weighted sum of the inputs of the hidden layer in the output layer neuron:

(5) $$v\left(n\right)=\sum_{i=1}^{p}w_{1i}\left(n\right)y_{i}\left(n\right)+c\left(n\right)$$

where *p* is the total number of hidden layer neurons, *c* is the bias and $\varphi_{o}$ is the activation function in the output layer. Next, the error signal is calculated. When the average squared error achieves a stopping criterion, the training is completed. This third stage is referred to as the forward pass computation. It is worth highlighting that in the forward pass computation, the weights and bias remain unaltered throughout the network. If the stopping criterion is not reached, the network training will continue to the next stage called the backward pass computation. This stage begins at the output layer by passing the error signals leftward through the network layer-by-layer and recursively computing the local gradient for each neuron as:

(6) $$\delta_{j}^{\left(l\right)}\left(n\right)=\left[\begin{array}{cc} e_{j}^{\left(L\right)}\left(n\right)\delta_{j}^{{}^{\prime }}\left(v_{j}^{\left(L\right)}\left(n\right)\right) & \text{for neuron j in output layer L}\\ \delta_{j}^{\prime }\left(v_{j}^{\left(L\right)}\left(n\right)\right)\sum_{k}\delta_{k}^{\left(l+1\right)}\left(n\right)w_{kj}^{\left(l+1\right)}\left(n\right) & \text{for neuron j in hidden layer l} \end{array}\right.$$

where $\delta_{j}^{\prime }\left(\cdot \right)$ denotes differentiation with respect to the argument. The synaptic weights and biases of the network in layer *l* according to the generalized delta rule are adjusted as:

(7) $$w_{ji}^{\left(l\right)}\left(n+1\right)=w_{ji}^{\left(l\right)}\left(n\right)+\alpha \left[w_{ji}^{\left(l\right)}\left(n-1\right)\right]+\eta \delta_{j}^{\left(l\right)}y_{i}^{l-1}\left(n\right)$$

where *η* is the learning-rate parameter and *α* is the momentum constant.

The fifth stage is the iteration. This stage presents new epochs of training patterns to iterate the forward and backward computations until the stopping criterion is met.

#### Training Patterns

The selection of the training patterns is a crucial process. The training patterns must contain data of the vehicle representative maneuvers, so as to characterize non-linear vehicle behavior.

As is mentioned in the previous section, the training pattern is formed by the input vector ($x\;=\;\left\{a_{ym},a_{xm},{\dot{\phi }}_{m},{\dot{\psi }}_{m}\right\}$) and the output ($d=\left\{\phi_{d}\right\}$). Each of the training patterns has been obtained from an experimentally-validated TruckSim vehicle model (see Section 3.2).

The maneuvers that have been conducted are a Double Lane Change (DLC) and Lane Change (LC) maneuvers (from 30 to 140 km/h), as well as J-turn maneuvers (from 20 to 45 km/h). All maneuvers have been simulated considering road friction coefficients of 0.3, 0.5 and 1. For DLC and LC maneuvers with a road friction coefficient of 0.3, the maximum speed had to be limited to 80 km/h. Higher speeds than 80 km/h caused the rollover of the vehicle. A summary of the training datasets is shown in Table 1.

### 2.2. Kalman Module

The Kalman module employs a discrete stochastic state-space form. The purpose of this module is to estimate the internal state of a linear dynamic system by means of a Kalman filter. The Kalman filter is a mathematical tool that is used for stochastic estimation from noisy sensor measurements. The real vehicle measurements include a substantial quantity of noise, as well as unobserved states in the system, which must be estimated. In this research, the unobserved state is the roll angle. The preliminary reconstruction of the roll angle obtained from the NN-based observer is used as a “pseudo-measurement” input to the Kalman filter. This previous calculation presents the advantage of considering the system non-linearities, thus providing good estimations, even though a linear vehicle model, represented as a state-space model, is used.

#### 2.2.1. State-Space Vehicle Model

**Figure 3:**
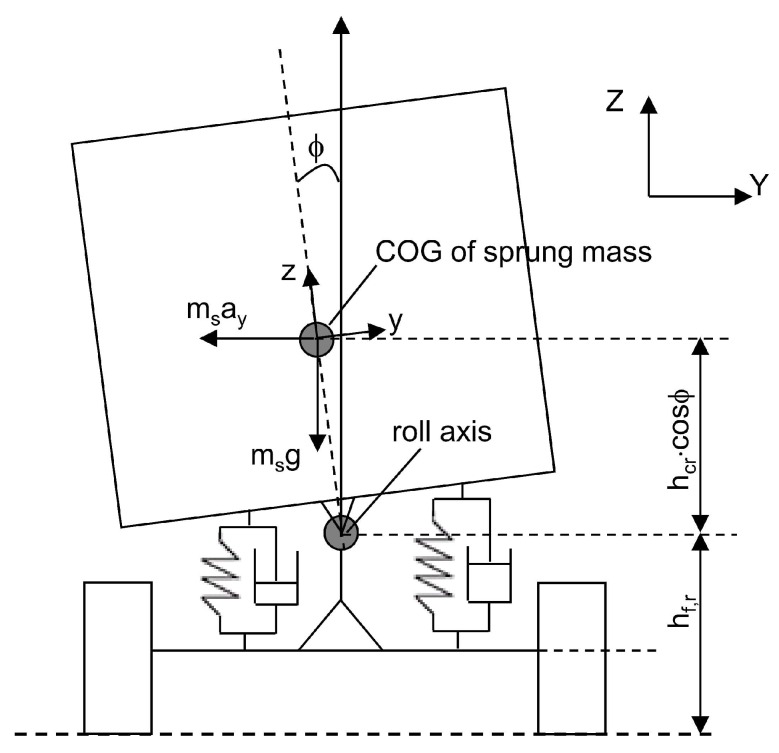
Schematic of the vehicle roll motion.

The dynamic vehicle model used in the Kalman filter algorithm is a linear model. When the vehicle model and measurement model equations are linear, the Kalman filter estimates the state vector recursively. An advantage of using linear systems is that they are easy to implement allowing the usage of the Kalman filter estimators in real time. For this reason, the dynamic model detailed in the estimation process is a 1-DOF vehicle model, which only represents vehicle roll motion. In Figure 3, the vehicle roll model is shown. The motion is described using a coordinate system (x, y, z) fixed in the vehicle. The vehicle roll angle, ϕ, is referenced from the vehicle’s vertical z-axis. It is assumed that the vehicle sprung mass rotates around the roll center of the vehicle. A detailed description of this model can be found in [10]. The differential equation obtained from the vehicle’s lateral dynamics can be written as:

(8) $$I_{xx}\ddot{\phi }+C_{R}\dot{\phi }+K_{R}\phi =m_{s}a_{y}h_{cr}+m_{s}h_{cr}g\sin\left(\phi \right)$$

where $I_{xx}$ is the moment of inertia of the sprung mass $m_{s}$ with respect to the roll axis, $C_{R}$ and $K_{R}$ denote respectively the total torsional damping and stiffness coefficients of the roll motion of the vehicle, $h_{cr}$ is the height of the sprung mass about the roll axis, *g* is the gravitational constant and $a_{y}$ is the lateral acceleration.

The lateral load transfer function can be obtained assuming that roll acceleration $\ddot{\phi }$ and velocity $\dot{\phi }$ are equal to zero. The steady-state equation for lateral load transfer applied to the left-hand side of the vehicle is given by:

(9) $$\Delta F_{zl}=-2\left(\frac{k_{f}}{e_{f}}+\frac{k_{r}}{e_{r}}\right)\phi -2m_{s}\frac{a_{y}}{l}\left(\frac{l_{r}h_{f}}{e_{f}}+\frac{l_{f}h_{r}}{e_{r}}\right)$$

where $h_{f}$ and $h_{r}$ are the heights of the front and rear roll centers, respectively; $k_{f}$ and $k_{r}$ are the front and rear roll stiffnesses, respectively; $e_{f}$ and $e_{r}$ are the front and rear vehicle tracks, respectively; and $l_{f}$ and $l_{r}$ are the distance from the COG (Center Of Gravity) to the front and rear axles, respectively. It must be noted that the lateral acceleration, $a_{y}$, used in Equations (8) and (9), is an inertial acceleration generated at the COG. Since the IMU provides a measurement of the acceleration due to the vehicle’s motion ($a_{ym}$) and due to gravitational acceleration (g), the lateral acceleration ($a_{y}$) can be computed as:

(10) $$a_{ym}=a_{y}\cos\left(\phi \right)+g\sin\left(\phi \right)$$

Assuming that the small roll angle approximation (i.e., sin(ϕ)≈ϕ and cos(ϕ)≈1) is valid, the measured lateral acceleration, $a_{ym}$, can be expressed as:

(11) $$a_{ym}=a_{y}+g\phi$$

In addition, assuming that the pitching and the bounding motion of sprung mass are neglected and the road bank angle is small, the vehicle roll rate can be expressed as:

(12) $$\dot{\phi }\approx {\dot{\phi }}_{m}$$

The vehicle model is represented as a continuous time state-space system as follows:

(13) $$\left\{\begin{array}{c} {\dot{x}}_{s}=Ax_{s}+w\\ y=Hx_{s}+v \end{array}\right.$$

where $x_{s}$ represents the state vector ${\left[\Delta F_{zl},a_{y},{\dot{a}}_{y},\phi ,\dot{\phi }\right]}^{T}$; A is the state evolution matrix; y is the measurement vector; ${\left[a_{ym},\phi ,{\dot{\phi }}_{m},\Delta F_{zl}\right]}^{T}$; H is the observation matrix; and w and v are the state disturbance and the observation noise vectors, respectively, that are assumed to be Gaussian, uncorrelated and zero mean:

(14) $$\begin{array}{c} w∼N(0,Q)\\ v∼N(0,R) \end{array}$$

where Q and R are the covariance matrices describing the second-order properties of state and measurement noise:

(15) $$Q=\left[\begin{array}{ccccc} 1000\left(N\right) & 0 & 0 & 0 & 0\\ 0 & 0.1\left(m/s^{-2}\right) & 0 & 0 & 0\\ 0 & 0 & 0.1\left(m/s^{-3}\right) & 0 & 0\\ 0 & 0 & 0 & 0.1\left(rad\right) & 0\\ 0 & 0 & 0 & 0 & 0.1\left(rad/s^{-2}\right) \end{array}\right]$$

(16) $$R=\left[\begin{array}{cccc} 0.01\left(m/s^{-2}\right) & 0 & 0 & 0\\ 0 & 0.01\left(rad\right) & 0 & 0\\ 0 & 0 & 0.01\left(rad/s^{-2}\right) & 0\\ 0 & 0 & 0 & 100\left(N\right) \end{array}\right]$$

R depends on sensor quality (the yaw and roll rates) and the lateral load transfer and pseudo-roll angle estimator quality. Q is often unknown and is tuned depending the developed model. Q and R are assumed time invariant and diagonal for simplicity reason.

According to the chosen state-space vector and measurements, the matrices A and H are defined as:

(17) $$\begin{array}{cc} A=\left[\begin{array}{ccccc} 0 & 0 & -2\frac{m_{s}}{l}\left(\frac{l_{r}h_{f}}{e_{f}}+\frac{l_{f}h_{r}}{e_{r}}\right) & 0 & -2\left(\frac{k_{f}}{e_{f}}+\frac{k_{r}}{e_{r}}\right)\\ 0 & 0 & 1 & 0 & 0\\ 0 & 0 & 0 & 0 & 0\\ 0 & 0 & 0 & 0 & 1\\ 0 & m_{s}\frac{h_{cr}}{I_{xx}} & 0 & \frac{m_{s}gh_{cr}-K_{R}}{I_{xx}} & -\frac{C_{R}}{I_{xx}} \end{array}\right] & H=\left[\begin{array}{ccccc} 0 & 1 & 0 & g & 0\\ 0 & 0 & 0 & 1 & 0\\ 0 & 0 & 0 & 0 & 1\\ 1 & 0 & 0 & 0 & 0 \end{array}\right] \end{array}$$

In order to operate with the sensor data, the discrete state-space system is obtained using the first order approximation of Euler ${\dot{x}}_{k-1}=\frac{x_{k}-x_{k-1}}{T_{s}}$, where $T_{s}$ is the sampling time. Therefore, the discrete system can be expressed as:

(18) $$\begin{array}{c} x_{s,k}=A_{d}x_{s,k-1}+w_{k}\\ y_{k}=Hx_{s,k}+v_{k} \end{array}$$

where $x_{s,k}=\left[\Delta F_{zl,k},a_{y,k},{\dot{a}}_{y,k},\phi \right]$, and the matrix $A_{d}$ can be expressed as:

(19) $$A_{d}=\left[\begin{array}{ccccc} 1 & 0 & -2T_{s}\frac{m_{s}}{l}\left(\frac{l_{r}h_{f}}{e_{f}}+\frac{l_{f}h_{r}}{e_{r}}\right) & 0 & -2T_{s}\left(\frac{k_{f}}{e_{f}}+\frac{k_{r}}{e_{r}}\right)\\ 0 & 1 & 1 & 0 & 0\\ 0 & 0 & 1 & 0 & 0\\ 0 & 0 & 0 & 1 & T_{s}\\ 0 & T_{s}m_{s}\frac{h_{cr}}{I_{xx}} & 0 & T_{s}\frac{m_{s}gh_{cr}-K_{R}}{I_{xx}} & 1+T_{s}\frac{C_{R}}{I_{xx}} \end{array}\right]$$

#### 2.2.2. Kalman Filter Algorithm

In this work, a Linear Kalman Filter (LKF) algorithm was used to estimate the vehicle state. The LKF is summarized in the following recursive equations:

1. Time update
  Prediction of state and covariance:
    (20) $${\bar{x}}_{s,k|k-1}=A_{d}{\bar{x}}_{s,k-1|k-1}$$
    (21) $$P_{s,k|k-1}=A_{d}P_{s,k-1|k-1}A_{d}^{T}+Q_{s}$$
2. Measurement update:
  Kalman gain:
    (22) $$K_{s,k}=P_{s,k|k-1}H^{T}{\left[HP_{s,k|k-1}H^{T}+R_{s}\right]}^{-1}$$
  State and covariance estimation:
    (23) $${\bar{x}}_{s,k|k}={\bar{x}}_{s,k|k-1}+K_{s,k}\left[y_{measured}-H{\bar{x}}_{s,k|k-1}\right]$$
    (24) $$P_{s,k|k}=\left[I-K_{s,k}H\right]P_{s,k|k-1}$$

The vector $y_{measured}={\left[a_{ym},\phi ,\dot{\phi },\Delta F_{zl}\right]}^{T}$ contains sensor data, such as the lateral acceleration, $a_{ym}$, and the roll rate, $\dot{\phi }$, and pseudo-measurements, such as the lateral load transfer, $\Delta F_{zl}$, calculated by Equation (9), and roll angle, ϕ. In order to prove the effectiveness of the proposed method, the pseudo-roll angle is computed in two different ways: (1) considering the suspension deflection, Equation (1); (2) considering the proposed NN estimator.

## 3. Results and Discussion

In this section, firstly, simulation results are presented to prove the effectiveness of the estimator proposed for different severe maneuvers and road conditions. Secondly, the proposed estimator is validated by means of experimental results using a real vehicle.

### 3.1. Experimental Vehicle Setup

**Figure 4:**
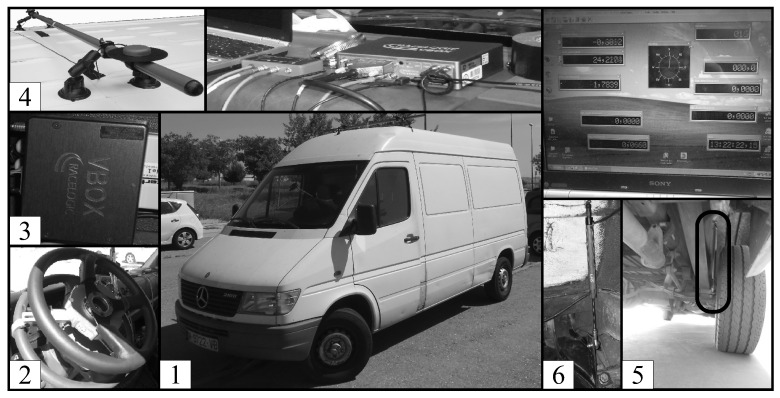
Experimental setup on a test vehicle. (1) Mercedes Sprinter. (2) Steering angle sensor MSW 250 Nm. (3) Vbox 3i dual antenna data logger. (4) GPS antennas. (5) LVDT MNT potentiometer. (6) SA-LP075 potentiometer.

The vehicle used for this research was a Mercedes Sprinter, as shown in Figure 4. The vehicle was equipped with a steering angle sensor MSW 250 Nm from Kistler, a Vbox 3i dual antenna data logger, an IMU and two GPS antennas from Racelogic. The IMU was mounted on the vehicle base, close to its COG. The two antennas were mounted at 90° to the true heading of the vehicle and on the roof, in order to accurately measure the roll angle. The roll angle value was used to validate the proposed estimator. Suspension deflection was experimentally measured by means of two linear potentiometers, Type SA-LP075 from 2D-Data, recording data from for the front suspension, and two sensors, Type LVDT MTN from Monitran, for the rear suspension.

The installed sensors provided measurements of the steering wheel, *δ*, the lateral acceleration, $a_{ym}$, the longitudinal acceleration, $a_{x}$, the vehicle longitudinal speed, $V_{x}$, the yaw rate, $\dot{\psi }$, the roll rate, $\dot{\phi }$, the roll angle, ϕ, the front left suspension deflection, $\Delta_{11}$, the front right suspension deflection, $\Delta_{12}$, the rear left suspension deflection, $\Delta_{21}$, and the rear right suspension deflection, $\Delta_{22}$.

### 3.2. Experimental Adjustment of Vehicle Model Parameters

The TruckSim simulation vehicle model was validated using experimental results from the real vehicle. TruckSim software [23] is a widely-used simulation software in the automotive industry that combines traditional and modern multi-body vehicle dynamics based on parametric modeling. One of the main advantages of using a simulated vehicle model is the ability to perform different types of vehicle maneuvers that attempt to avoid possible accidents under different road conditions. In addition, simulation models guarantee test reproducibility.

**Figure 5:**
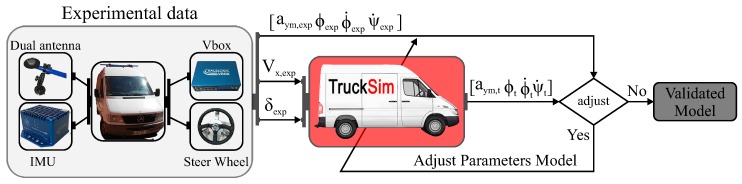
Scheme of the vehicle model design.

The schema for the vehicle model validation is shown in Figure 5. The lateral acceleration ($a_{ym,t}$), the roll rate (${\dot{\phi }}_{t}$), the roll angle ($\phi_{t}$) and the yaw rate (${\dot{\psi }}_{t}$) obtained from the simulated model were compared with experimental data. The model parameters were adjusted by trial and error according to the differences between the experimental and simulation data.

**Figure 6:**
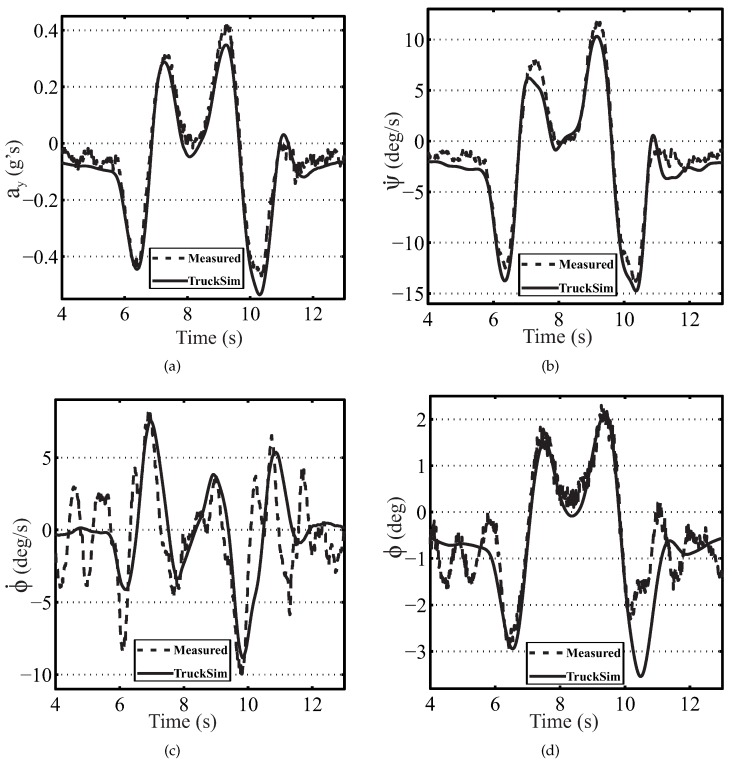
DLC maneuvers at 70 km/h for the experimental adjustment of vehicle model parameters (solid line: TruckSim; dashed line: measured): (**a**) lateral acceleration, (**b**) yaw rate, (**c**) roll rate and (**d**) roll angle.

The performance of the vehicle model was proven in both the DLC and LC maneuvers with vehicle speeds of 50 and 70 km/h on dry pavement. Figure 6 shows the comparative results for the TruckSim simulation vehicle model using the experimental data obtained during a DLC maneuver for the real vehicle traveling at 70 km/h. The figure indicates excellent agreement between the simulation and the experimental results.

In addition to the graphical evidence of the effectiveness of the proposed simulation model, a quantitative analysis that took into consideration the error of the different maneuvers was computed. The following equation was used to represent the norm error as a function of time [24]:

(25) $$E_{t}=\frac{\varepsilon_{t}}{\sigma_{t}}$$

where:

(26) $$\begin{array}{c} \varepsilon_{t}=\int_{0}^{T}{\left(\lambda_{Exp}-\lambda_{Trucksim}\right)}^{2}\\ \sigma_{t}=\int_{0}^{T}{\left(\lambda_{Exp}-\mu_{Exp}\right)}^{2} \end{array}$$

where $\lambda_{Exp}$ and $\lambda_{Trucksim}$ represent the measured and simulated lateral acceleration, yaw and roll rates and roll angle, respectively, and $\mu_{Exp}$ is the mean value of the lateral acceleration, yaw rate and roll angle obtained from the real vehicle during time T.

For the DLC at 70 km/h (see Figure 6), the norm error of the lateral acceleration, the yaw and roll rates and the roll angle were 0.221, 0.210, 0.792 and 0.522, respectively. The norm and maximum errors are provided in Table 2 for DLC at 50 km/h and LC at 50 and 70 km/h. From the results, we can conclude that the created vehicle model accurately represents the real vehicle.

Table 3 shows the adjusted parameters for the state-space vehicle model.

### 3.3. Simulated Validation

To prove the effectiveness of the proposed roll angle estimator based on NN and the Kalman filter, two severe maneuvers, such as the sine sweep and slalom, were conducted. The former was performed at 50 and 70 km/h on a road surface with friction coefficients of 0.7 and 0.3, respectively. The latter was conducted at 35 km/h for the same friction coefficients. In order to analyze the effect of the sensor measurement’s noise on the estimation of the roll angle, Gaussian noises with zero mean and variances of 0.01 m/s${}^{2}$, 0.01 m/s${}^{2}$, 0.01°/s and 0.01°/s were added to $a_{ym}$ (lateral acceleration), $a_{x}$ (longitudinal acceleration), $\dot{\phi }$ (roll rate) and $\dot{\psi }$ (yaw rate), respectively.

**Figure 7:**
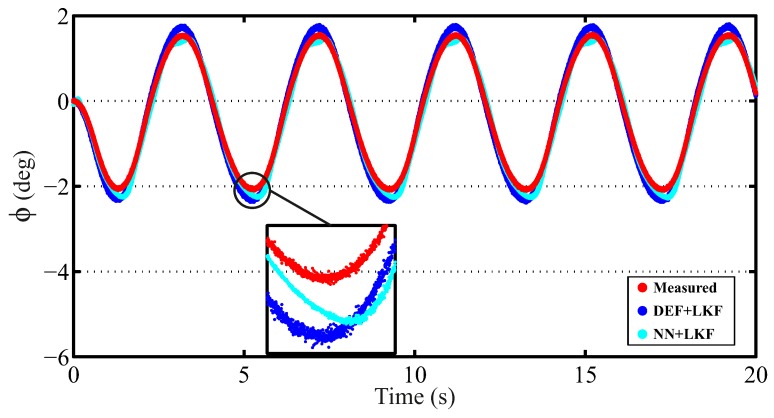
Simulation results for a slalom maneuver at 35 km/h with a friction coefficient of 0.3 (red points: measured; blue points: Deflection (DEF) + Linear Kalman Filter (LKF); cyan points: NN + LKF).

**Figure 8:**
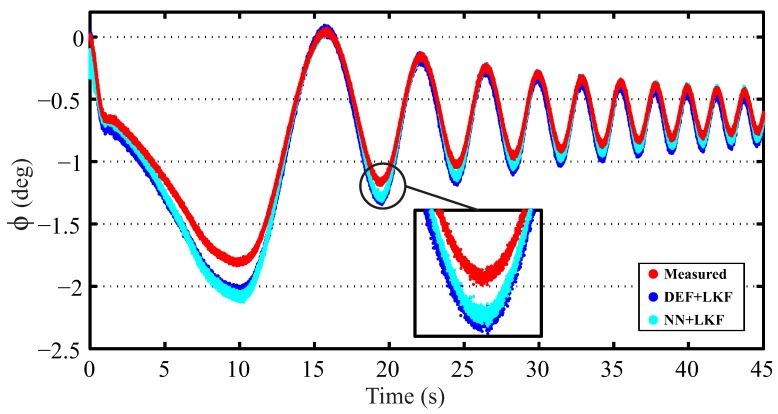
Simulation results for a sine sweep maneuver at 70 km/h with a friction coefficient of 0.3 (red points: measured; blue points: DEF + LKF; cyan points: NN + LKF).

Figure 7 shows the comparative results of the Deflection + LKF-based observer (DEF + LKF) and NN + LKF-based observer for a slalom maneuver at 35 km/h with a friction coefficient of 0.3. Figure 8 shows the comparative results of the DEF + LKF-based observer and NN + LKF-based observer for a sine sweep maneuver at 70 km/h with a friction coefficient of 0.3. These figures demonstrate that the proposed observer based on NN yields a better behavior than the observer based on the suspension deflection.

The norm and maximum errors are provided in Table 4. The norm errors for the NN + LKF-based estimator are smaller than those from DEF + LKF-based estimator. The differences in maximum errors are higher in the cases for which the friction coefficient is 0.3. However, the maximum difference is 0.086°, which is negligible.

The proposed method provides better results in 100% of the analyzed simulated cases (four cases) for the norm error; whereas, a 50% success rate is obtained for the maximum error compared with the DEF + LKF method.

### 3.4. Experimental Validation

The performance of the proposed estimator was verified for a real vehicle traveling at different speeds on dry pavement under different maneuvers.

**Figure 9:**
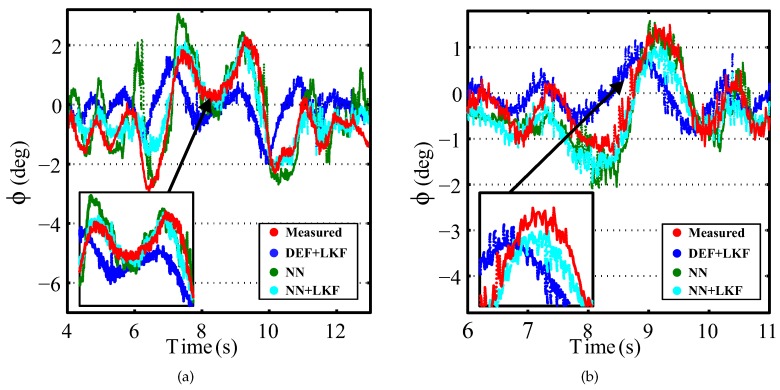
Experimental results for a vehicle traveling at 70 km/h on dry pavement. (red points: measured; blue points: DEF + LKF; green points: NN; cyan points: NN + LKF): (**a**) DLC maneuver and (**b**) LC maneuver.

Figure 9 shows the results for DLC and LC maneuvers for a real vehicle traveling at 70 km/h. Table 5 shows the norm and maximum errors for DLC and LC at speeds of 50 and 70 km/h. In all cases, the norm error of the proposed estimator is smaller than the estimator based on suspension deflection. However, when the vehicle is traveling at 50 km/h, the maximum error of the NN + LKF-based observer is greater for both maneuvers. Nevertheless, the maximum difference is 0.2°, which is acceptable taking into account that the maximum measured roll angle at 50 km/h is about 2°. In order to prove the necessity of incorporating a LKF in the NN estimator, the results for a single NN estimator are also shown. In the majority of cases (three out of four cases), the NN + LKF-based estimator improves the roll angle estimation.

**Figure 10:**
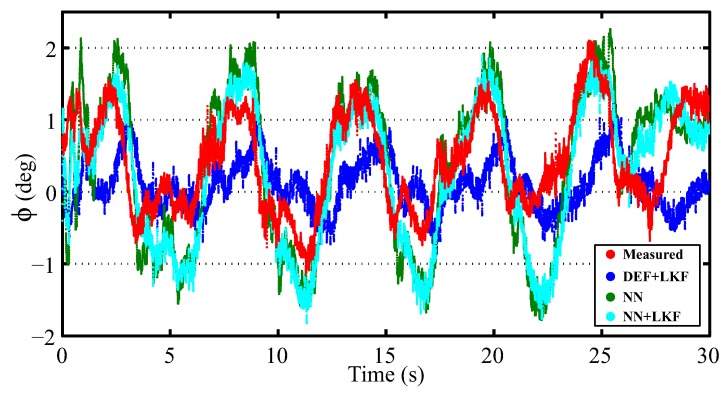
Experimental results for slalom maneuver (red points: measured; blue points: DEF + LKF; green points: NN; cyan points: NN + LKF).

Additionally, the proposed estimator was evaluated when the vehicle is traveling at about 45 km/h under a slalom maneuver. The results are depicted in Figure 10. The norm and maximum errors of the NN + LKF estimator are 0.921 and 2.070°, respectively; whereas, the norm and maximum errors for the NN-based and DEF + LKF-based estimators are [1.040; 2.232°] and [1.181; 2.081°], respectively. In this case, the proposed estimator yields lower errors.

**Figure 11:**
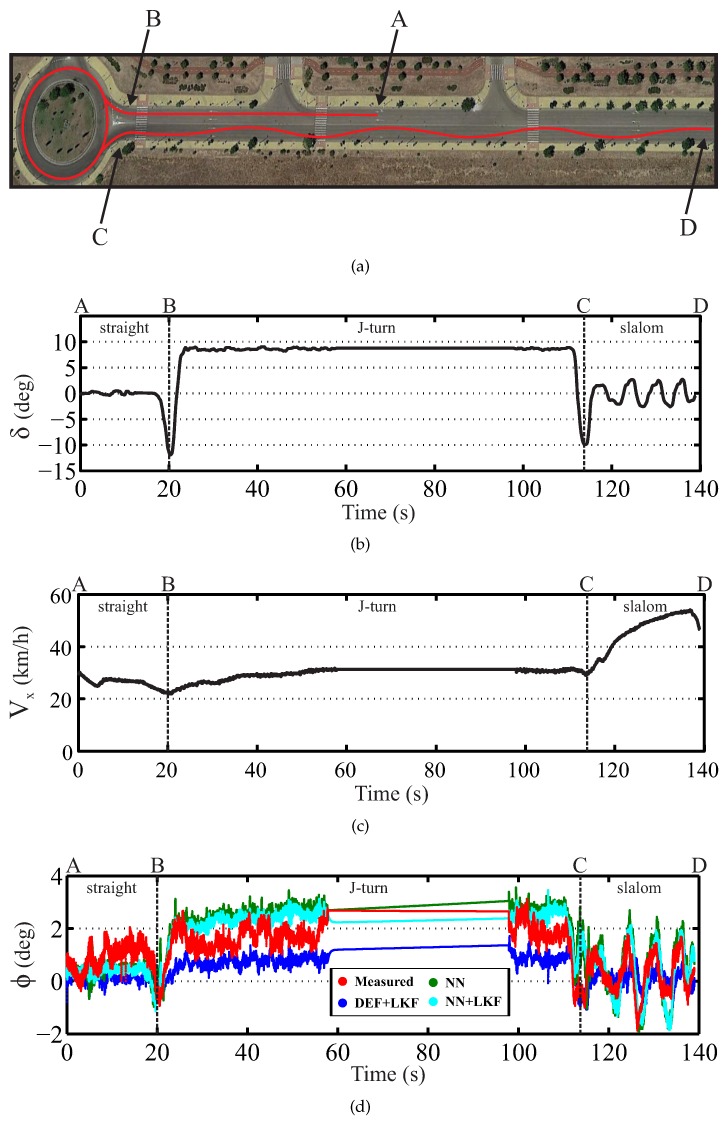
Experimental results for J-turn and slalom maneuvers (red points: measured; blue points: DEF + LKF; green points: NN; cyan points: NN + LKF): (**a**) the real environment and the vehicle trajectory, (**b**) the steering wheel angle profile, (**c**) the longitudinal vehicle speed profile and (**d**) the real and estimated roll angle.

**Figure 12:**
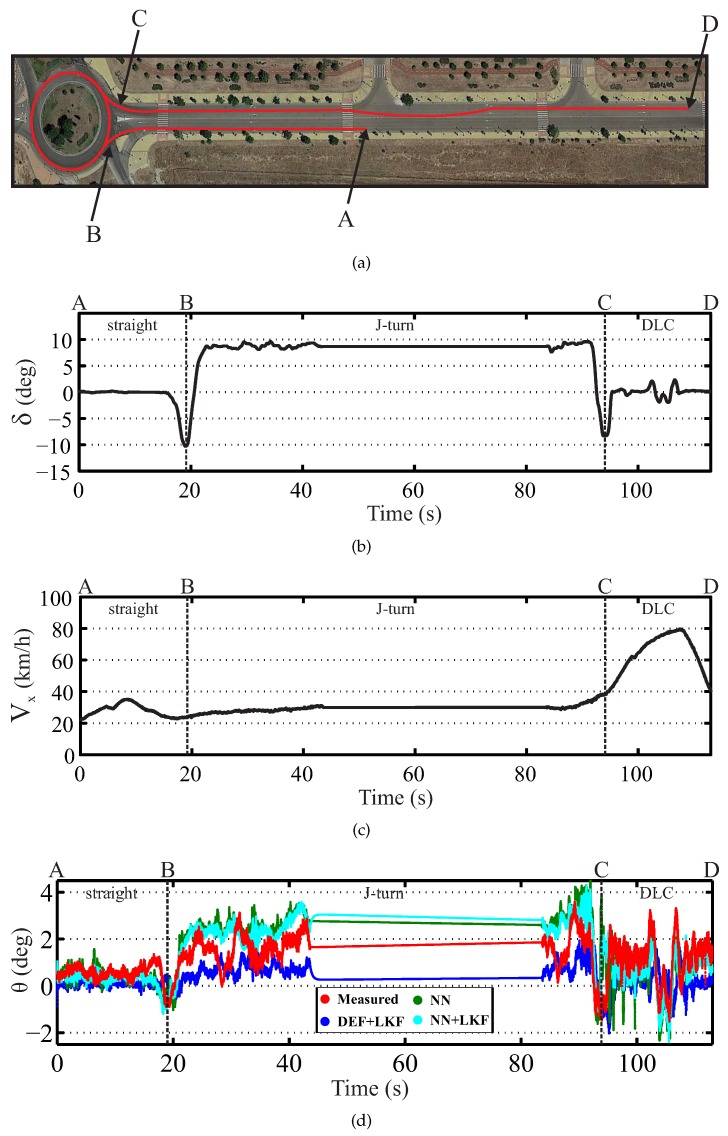
Experimental results for J-turn and DLC maneuvers (red points: measured; blue points: DEF + LKF; green points: NN; cyan points: NN + LKF): (**a**) the real environment and the vehicle trajectory, (**b**) the steering wheel angle profile, (**c**) the longitudinal vehicle speed profile and (**d**) the real and estimated roll angle.

Finally, two tests were performed with a combination of different maneuvers, as depicted in Figure 11 and Figure 12. These figures show the real environment and the vehicle trajectory, the steering wheel angle profile, the longitudinal vehicle speed profile and the real and estimated roll angle.

The first test corresponds to a real vehicle traveling on dry pavement under a J-turn and slalom maneuvers and the second one to the same vehicle under a J-turn and DLC. Figure 11a shows a J-turn and slalom maneuvers, while Figure 12a depicts a J-turn and DLC maneuvers. Table 6 and Table 7 show the norm and maximum errors. The proposed NN + LKF-based estimator proves to be better than the suspension deflection and single NN estimators, for both the norm and maximum errors.

The proposed method provides better results in 100% of the analyzed experimental cases (10 cases) for the norm error; whereas, an 80% success rate is obtained for the maximum error compared with the DEF + LKF method. In the worst case, the maximum difference between the DEF + LKF and NN + LKF is 0.197°, which is negligible.

## 4. Conclusions

This article presents a novel estimator based on sensor fusion, which combines NN and LKF in order to estimate the vehicle roll angle. The proposed vehicle roll angle estimator architecture is formed by two modules: the NN module and the Kalman module. The NN module estimates a pseudo-roll angle through data, such as the lateral and longitudinal accelerations, the yaw and roll rates. The pseudo-roll angle is passed on to the Kalman module in order to filter noise and minimize the norm and maximum errors. The proposed NN + LKF-based estimator takes into account the vehicle motion nonlinearities. The main advantages of the proposed method are that only a single IMU is needed, and no additional suspension deflection sensors are required.

The NN + LKF-based estimator was validated with simulated and experimental results. The simulation analysis has proven the effectiveness of the proposed estimator in severe maneuvers with low and medium friction coefficients (0.3 and 0.7). In addition, experimental tests, such as, slalom, J-turn and DLC maneuvers, were conducted on dry pavement. For the overall analyzed tests, simulated and experimental ones, the proposed method yielded better results in 100% for the norm error compared with the DEF + LKF method; whereas, a 71% success rate was obtained for the maximum error, reaching an 80% success rate for experimental tests. In the worst case, the maximum difference between the DEF + LKF and NN + LKF is 0.197°, which is negligible.

Experimental results also show the necessity of including the LKF in the NN estimator in order to filter noise and, therefore, to improve the roll angle estimation.

## Figures and Tables

**Table 1:** Training datasets for NN, DLC, Double Lane Change, LC, Lane Change.

Road Friction Coefficients	Maneuvers	Speeds (km/h)
0.5–1	DLC	30, 40, 50, 60, 70, 80, 90, 100, 110, 120, 130, 140
	LC	
0.3	DLC	30, 40, 50, 60, 70, 80
	LC	
0.3–0.5–1	J-turn	20, 25, 30, 35, 40, 45

**Table 2:** Norm and maximum errors for the DLC and LC maneuvers.

	DLC at 50 km/h		DLC at 70 km/h		LC at 50 km/h		LC at 70 km/h	
	$E_{t}$	$E_{\max}$	$E_{t}$	$E_{\max}$	$E_{t}$	$E_{\max}$	$E_{t}$	$E_{\max}$
$a_{ym}$ ($g^{\prime }s$)	0.130	0.080	0.221	0.138	0.138	0.035	0.418	0.050
$\dot{\psi }$ (°/s)	0.114	1.748	0.210	2.791	0.109	1.365	0.176	0.827
$\dot{\phi }$ (°/s)	0.696	4.023	0.792	8.240	0.587	3.322	1.134	5.115
*ϕ* (°)	0.678	2.206	0.522	2.068	0.621	0.675	1.279	0.273

**Table 3:** State-space vehicle model parameters, COG, Center Of Gravity.

Symbol	Description	Value	Unit
$C_{R}$	Total torsional damping of the suspension	54,447.87	Nms/rad
$e_{f}$	Front vehicle track	1.638	m
$e_{r}$	Rear vehicle track	1.630	m
$h_{cr}$	Height of the sprung mass about the roll axis	0.980	m
$h_{f}$	Height of the front roll center	0.348	m
$h_{r}$	Height of the rear roll center	0.348	m
$I_{xx}$	Roll inertia	500	kg·m${}^{2}$
$k_{f}$	Front roll stiffness	371,530.334	Nm/rad
$k_{r}$	Rear roll stiffness	371,530.334	Nm/rad
$K_{R}$	Total torsional stiffness of the suspension	743,060.669	Nm/rad
$l_{f}$	Distance from front tire to COG	1.51	m
$l_{r}$	Distance from rear tire to COG	2.04	m
$m_{s}$	sprung mass	1700	kg

**Table 4:** Norm and maximum errors for roll angle estimators for sine sweep and slalom maneuvers.

	SLA-35-0.7		SLA-35-0.3		SS-50-0.7		SS-70-0.3	
	$E_{t}$	$E_{\max}$	$E_{t}$	$E_{\max}$	$E_{t}$	$E_{\max}$	$E_{t}$	$E_{\max}$
	(-)	(°)	(-)	(°)	(-)	(°)	(-)	(°)
DEF + LKF	0.221	0.158	0.250	0.279	0.122	0.302	0.129	0.295
NN + LKF (*)	0.093	0.152	0.230	0.365	0.078	0.282	0.099	0.349

(*) Proposed; SLA-35-0.7: Slalom at 35 km/h and friction coefficient 0.7; SLA-35-0.3: Slalom at 35 km/h and friction coefficient 0.3; SS-50-0.7: Sine sweep at 50 km/h and friction coefficient 0.7; SS-70-0.3: Sine sweep at 70 km/h and friction coefficient 0.3.

**Table 5:** Norm and maximum errors for roll angle estimators for the DLC and LC maneuvers.

	DLC at 50 km/h		DLC at 70 km/h		LC at 50 km/h		LC at 70 km/h	
	$E_{t}$	$E_{\max}$	$E_{t}$	$E_{\max}$	$E_{t}$	$E_{\max}$	$E_{t}$	$E_{\max}$
	(-)	(°)	(-)	(°)	(-)	(°)	(-)	(°)
DEF + LKF	0.912	1.526	1.038	2.786	1.206	0.903	2.087	0.477
NN	0.806	2.602	0.692	1.620	0.726	0.963	0.849	0.962
NN + LKF (*)	0.547	1.723	0.446	1.841	0.478	0.961	0.964	0.276

(*) Proposed.

**Table 6:** Norm and maximum errors for roll angle estimators for J-turn and slalom maneuvers.

	Total Sector		J-Turn Sector		Slalom Sector	
	$E_{t}$	$E_{\max}$	$E_{t}$	$E_{\max}$	$E_{t}$	$E_{\max}$
	(-)	(°)	(-)	(°)	(-)	(°)
NN	0.757	2.071	1.174	1.799	1.026	1.664
DEF + LKF	1.081	2.495	1.571	2.220	1.073	1.980
NN + LKF	0.655	1.689	0.969	1.689	0.879	1.591

**Table 7:** Norm and maximum errors for roll angle estimators for J-turn and DLC maneuvers.

	Total Sector		J-Turn Sector		DLC Sector	
	$E_{t}$	$E_{\max}$	$E_{t}$	$E_{\max}$	$E_{t}$	$E_{\max}$
	(-)	(°)	(-)	(°)	(-)	(°)
NN	1.347	3.680	1.547	1.572	0.836	2.811
DEF + LKF	1.687	4.196	1.646	2.663	1.461	3.752
NN + LKF	1.323	2.307	1.440	1.501	0.753	2.093
